# Preventive effect of celecoxib use against cancer progression and occurrence of oral squamous cell carcinoma

**DOI:** 10.1038/s41598-017-06673-3

**Published:** 2017-07-24

**Authors:** Shang-Lun Chiang, Bharath Kumar Velmurugan, Chia-Min Chung, Shu-Hui Lin, Zhi-Hong Wang, Chun-Hung Hua, Ming-Hsui Tsai, Tzer-Min Kuo, Kun-Tu Yeh, Pei-Ying Chang, Yi-Hsin Yang, Ying-Chin Ko

**Affiliations:** 1 Environment-Omics-Diseases Research Centre, China Medical University Hospital, Taichung, Taiwan; 2 Department of Health Risk Management, College of Public Health, China Medical University, Taichung, Taiwan; 3 Faculty of Applied Science, Ton Duc Thang University, Ho Chi Minh City, Vietnam; 4 Graduate Institute of Clinical Medical Science, China Medical University, Taichung, Taiwan; 5 Department of Surgical Pathology, Changhua Christian Hospital, Changhua, Taiwan; 6 Department of Otorhinolaryngology, China Medical University Hospital, Taichung, Taiwan; 7 Department of Oral and Maxillofacial Surgery, China Medical University Hospital, Taichung, Taiwan; 8 School of Pharmacy and Cancer Centre, Kaohsiung Medical University, Kaohsiung, Taiwan

## Abstract

Overexpression of cyclooxygenase-2 in oral cancer increases lymph node metastasis and is associated with a poor prognosis. The potential of celecoxib (CXB) use is reported in cancer treatment by inhibiting proliferation through apoptosis, but the effects on the epithelial-mesenchymal transition (EMT) and cancer cell mobility remain unclear. We performed a preclinical study and population-based study to evaluate CXB use in the prevention of oral cancer progression and occurrence. The *in*-*vitro* findings showed that CXB is involved in the inhibition of EMT and cell mobility through blocking transcription factors (Slug, Snail and ZEB1), cytoplasmic mediators (focal adhesion kinase (FAK), vimentin and β-catenin), cell adhesion molecules (cadherins and integrins), and surface receptors (AMFR and EGFR). The murine xenograft model showed a 65% inhibition in tumour growth after a 5-week treatment of CXB compared to placebo. Xenograft tumours in placebo-treated mice displayed a well-to-moderate/moderate differentiated SCC grade, while those from CXB-treated mice were well differentiated. The expression levels of membrane EGFR, and nuclear FAK, Slug and ZEB1 were decreased in the xenograft tumours of CXB-treated mice. A retrospective cohort study showed that increasing the daily dose and medication time of CXB was associated with oral cancer prevention. The findings provide an alternative prevention strategy for oral cancer development with CXB use.

## Introduction

Oral squamous cell carcinoma (OSCC) is a head and neck cancer and a major cause of significant morbidity worldwide^1^. The high incidence and mortality highlight the importance of OSCC control through the early prevention of tumour initiation and progression. More than 80% of head and neck premalignant or malignant tissues had overexpression of cyclooxygenase-2 (COX-2)^2, 3^. In murine models, evidence showed that COX-2 overexpression was sufficient to induce tumorigenesis^4, 5^. In addition, overexpression of COX-2 signalling is associated with lymph node metastasis in head and neck cancer patients^6^. The clinical practice guidelines for the management and treatment of OSCC are often according to the pathological TNM staging and clinical risk features, including perineural invasion, lymphovascular permeation and extracapsular nodal spread. These risk features have commonly been used as the major prognostic factors in OSCC^7, 8^. The presence of cervical node metastases is strongly associated with higher recurrence rates as well as shorter survival rates, especially in OSCC patients with extremely high COX-2 levels^9^.

The initial events in cancer cell migration and scattering are alterations in cell adhesion, including cell-cell junctions and cell-extracellular matrix (ECM) interactions. The ordered structure of epithelial and endothelial tissues mostly involves E-cadherin and N-cadherin^10, 11^. In clinical pathology, loss of E-cadherin is often accompanied by increasing levels of N-cadherin and vimentin in cancer cells, which are characteristic of EMT progression^12^. E-cadherin transcription is well known to be suppressed by the transcription factors Slug, Snail and Twist during EMT programs, while N-cadherin is significantly elevated by Snail and Twist^13–15^. Exogenous prostaglandin E2 (PGE_2_) treatment led to up-regulation of Snail and ZEB1 and down-regulation of E-cadherin in both mRNA and protein expression^16^. Moreover, a decreased epithelial E-cadherin level was found to be associated with increased PGE_2_ synthesis through prostaglandin EP2 receptors during SCC progression^17^. These findings indicate that abnormal COX-2/PGE_2_ overexpression is involved in cancer development, and whether it could be reversible by prescribing medication interventions of COX-2 inhibitors in OSCC patients remains to be investigated.

CXB is a selective COX-2 inhibitor with reduced adverse effects in clinical practice and is used for anti-inflammation therapy in several diseases, such as rheumatoid arthritis, osteoarthritis, ankylosing spondylitis, juvenile arthritis, menstrual pain, and chronic pain. Remarkably, special application of CXB is approved for use in early cancer prevention in subjects with familial adenomatous polyposis^18^. Many studies are still trying to determine the efficacy of CXB alone or in combined therapeutic strategies in the treatment of several premalignant or malignant cancers by preclinical or clinical trials.

Of the various molecular mechanisms for CXB in anti-neoplastic progression, anti-angiogenesis activity through decreasing COX-2-induced VEGF production^6^ and anti-apoptosis effects through the inhibition of caspase signalling have been reported^19, 20^. In addition, the anti-EMT properties of CXB were demonstrated in human colon and bladder cancer cell lines^21, 22^. A poor outcome was observed in most OSCC patients with recurrence or nodal metastasis. In this study, we first attempt to determine the possible efficacy of CXB in inhibiting the OSCC cancer cell proliferation, mobility and EMT programs through several related signalling pathways using pre-clinical models. Subsequently, we perform a population-based study to evaluate the benefit of CXB use as an early chemopreventive strategy on the occurrence of OSCC development.

## Results

### Inhibition of OSCC growth, migration and invasion *in vitro*

**Figure 1:**
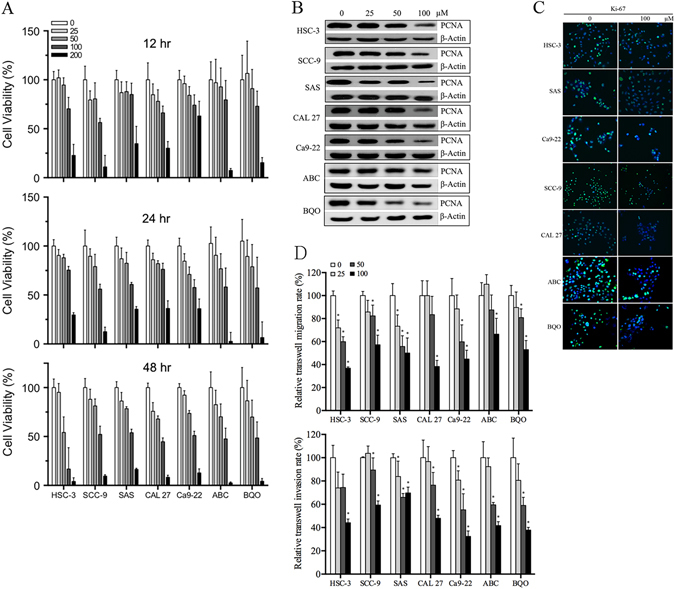
Inhibitory effect of CXB on OSCC tumour growth, migration and invasion in an *in vitro* model. (**A**) MTT-based cell viability assay, (**B**) immunoblotting of PCNA, (**C**) ICC-IF of Ki-67 (Ki-67 and DAPI showed green and blue fluorescence, respectively), and (**D**) cell migration ability was determined by the scratch (also refer Supplementary Fig. 2) and transwell assays, and invasion was analysed using a matrigel-transwell assay.

We performed 7 OSCC cells to test the CXB effect on inhibiting tumour cell growth. The karyotypes of primarily cultured BQO and ABC cells are shown in Supplementary Fig. 1, and both cells were determined without 13 high-risk HPV infections (RLU/Cut-off < 0.5). Most OSCC cells had significantly decreased viability after 24 h and 48 h of treatment with CXB (50/100 μM) compared with untreated cells (Fig. 1A). In addition, we observed that CXB inhibited OSCC cell proliferation in 24 h of treatment assessed by PCNA immunoblotting and Ki-67 (green fluorescence) ICC-IF assays (Fig. 1B and C). In both scratch-wound screening and the transwell validated migration assay, we found that 24 h treatment with CXB, particularly at 50 and 100 μM, had significant inhibitory effects on OSCC cell migration compared with untreated cells (Fig. 1D and Supplementary Fig. 2). Subsequently, the same treated conditions also showed a significant inhibitory effect in the OSCC invasion ability.

### Inhibitory signalling relative to proliferation, EMT programs and cell motility

**Figure 2:**
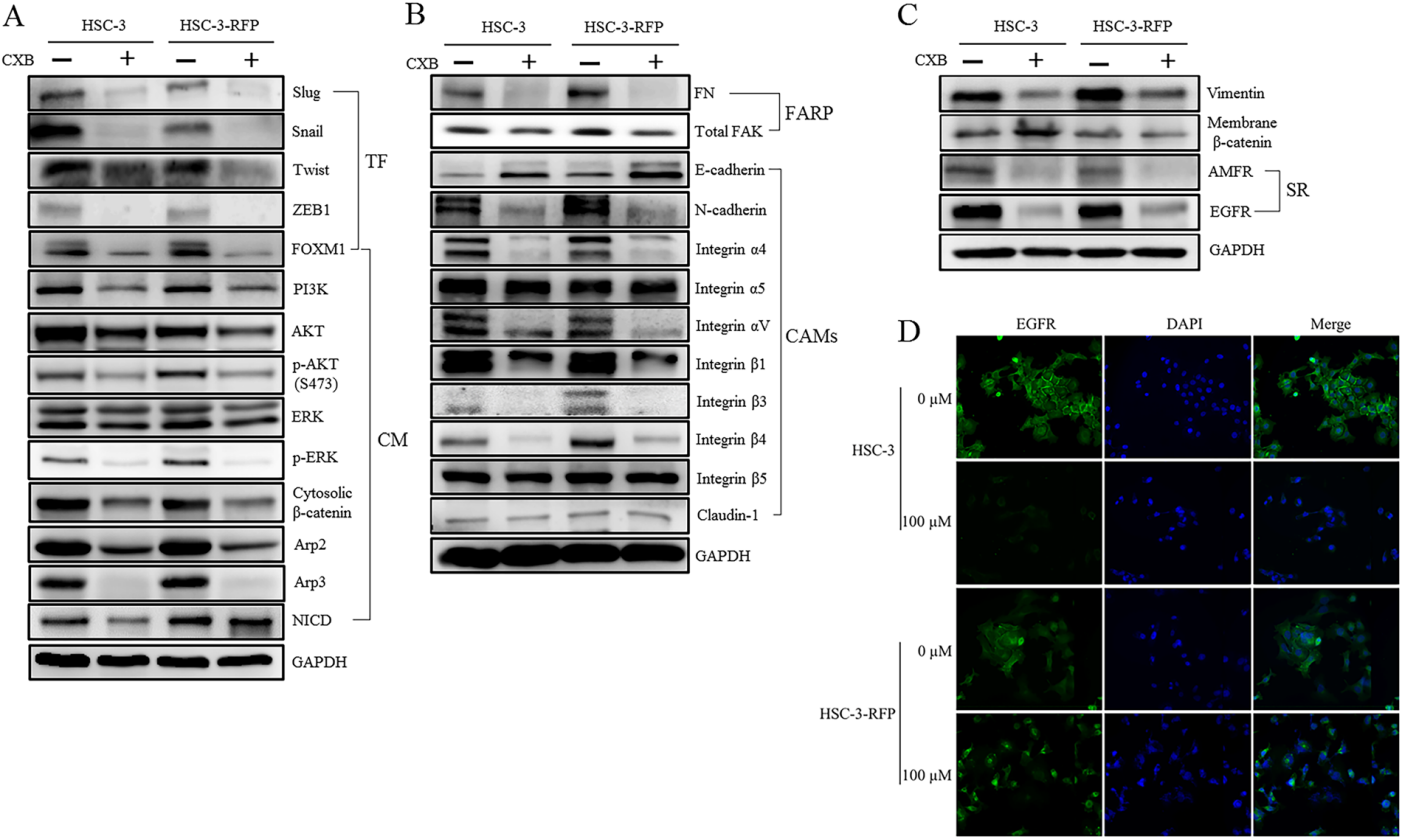
The molecular mechanism of CXB effect on OSCC proliferation, EMT programs and cancer cell motility. The expression level of selected markers of (**A**) nuclear transcriptional factors (TF) and cytoplasmic mediators (CM); (**B**) focal adhesion related proteins (FARP) and cell adhesion molecules (CAMs); (**C**) intermediate filament protein, membrane binding protein and surface receptors (SR) were determined between CXB-untreated and treated HSC-3 and HSC-3-RFP cells in an immunoblotting assay (also refer Supplementary Fig. 3); and (**D**) the inhibitory effect of CXB on EGFR (green fluorescence) was further validated by the ICC-IF assay.

Selected proliferation biomarkers, FOXM1 and cytoplasmic mediators [PI3K, AKT, p-AKT^Ser473^, ERK, p-ERK and NOTCH1 intracellular domain (NICD)], were examined for understanding the CXB inhibitory effect on the proliferation of HSC-3 and HSC-3-RFP cell lines. The expression levels of these proliferation markers were inhibited after 24 h of treatment with CXB (100 μM) (Fig. 2A and Supplementary Fig. 3A). Important biomarkers involved in the signal transduction of cancer cell migration and metastasis were also determined (Fig. 2A,B,C and Supplementary Fig. 3). The endogenous levels of nuclear transcription factors (Slug, Snail, Twist and ZEB1), cytoplasmic mediators (cytosolic β-catenin, Arp2 and Arp3), focal adhesion related proteins (FN and FAK), cell adhesion molecules (N-cadherin and integrin α4, α5, αV, β1, β3 and β4), intermediate filament protein (Vimentin), and surface receptors (autocrine motility factor receptor (AMFR) and EGFR) were inhibited following 24 h of CXB treatment in HSC-3 and HSC-3-RFP cells, while E-cadherin was induced. Integrin β5 and Claudin-1 had inconsistent expression levels after treatment in HSC-3 and HSC-3-RFP cells. The membrane β-catenin extracted from the total membrane protein of both cell lines was found to increase following treatment although it showed no statistical significance in HSC-3-RFP cells. Furthermore, the inhibitory effect of CXB in EGFR expression in both OSCC cell lines was validated by ICC-IF (Fig. 2D).

### CXB on *in vivo* OSCC growth in a murine xenograft tumour model

**Figure 3:**
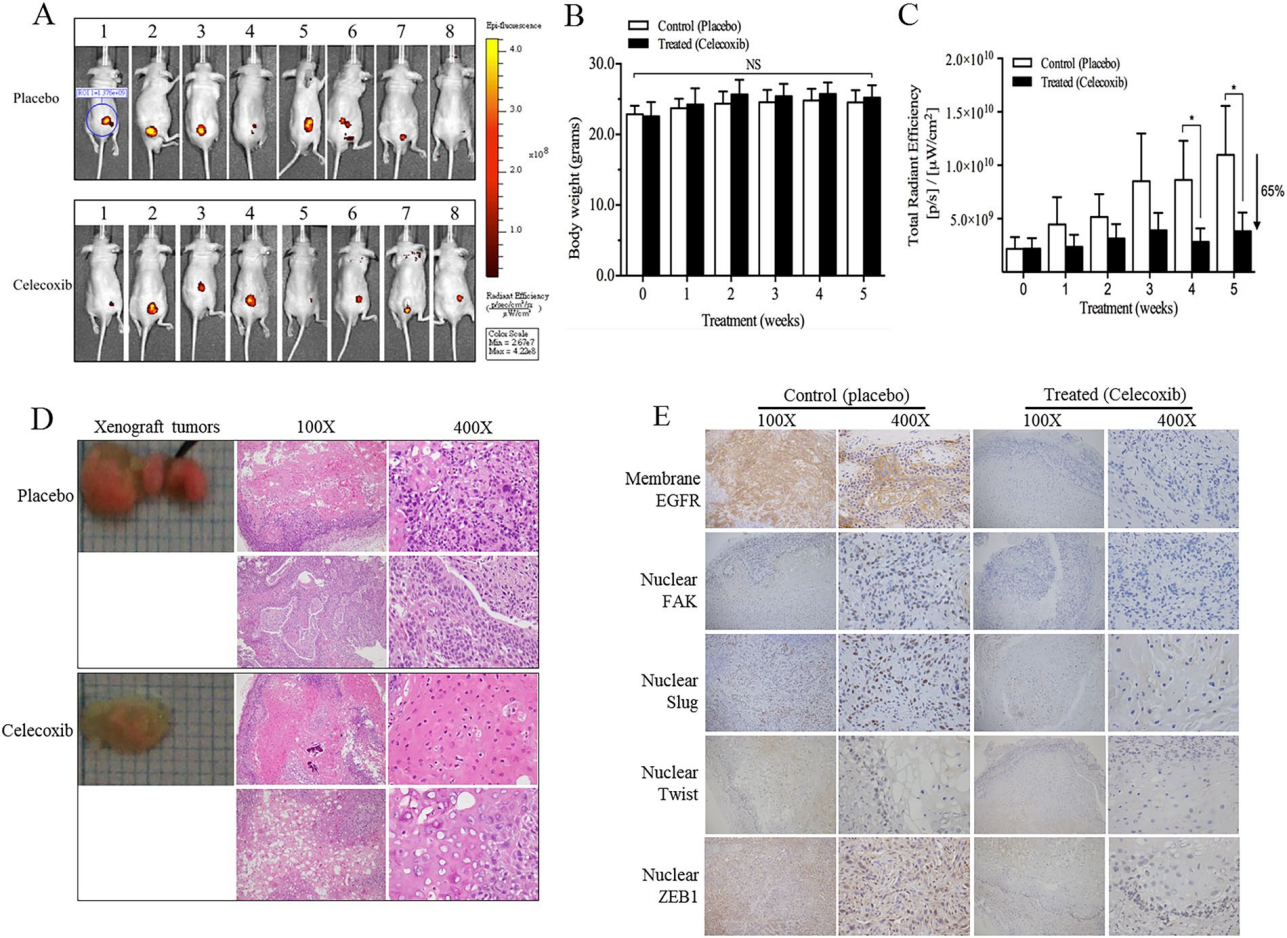
Effect of CXB in a murine xenograft model of OSCC. (**A**) The fluorescence signal of xenografted mice after 5-week treatment of CXB and placebo, respectively, was determined using an IVIS 2000 system. (**B**) Comparison of the murine body weight between CXB and placebo treatments. (**C**) Inhibition of xenograft tumour growth in the CXB-treated group compared with the placebo group. (**D**) Histological grade of excised xenograft tumours between CXB and placebo treated mice. (**E**) Measurement of the expression level of membrane EGFR and nuclear FAK, Slug, Twist and ZEB1 by IHC analysis (also refer Table 1).

After xenografting stable HSC-3-RFP cells in 5-week-old mice, all mice were followed up at approximately 6 weeks for tumour growth prior to grouping and CXB/placebo treatment. After 5-week treatments, we observed that the RFP signal intensity of xenograft tumour was lower in most mice treated with CXB than in those with placebo (Fig. 3A). In this murine model, no drug toxicity occurred in the gastrointestinal tract after 5-week treatments based on the observation of body weight between the CXB-treated and placebo groups (Fig. 3B). The fluorescent signal of xenograft tumours in CXB-treated mice had a significant decrease after more than 4 weeks of treatment, and it had approximately 65% tumour growth inhibition following 5 weeks of treatment compared with the placebo group (Fig. 3C). Two mice, 1 untreated (Placebo #8) and 1 treated (Celecoxib #5), were excluded from IHC analysis because it was difficult to excise the xenograft tumours although the low intensity of fluorescence emission was still visualized. The pathohistological status of excised xenograft tumours from placebo-treated mice revealed well-to-moderate or moderate differentiated SCC grade, while those from CXB-treated mice were well-differentiated (Fig. 3D). Compared to the placebo group, the expression levels of membrane EGFR, nuclear FAK and ZEB1 of xenograft tissue were significantly decreased in CXB-treated mice by IHC analyses using the Allred and IRS combinative scoring systems (p < 0.05) (Fig. 3E and Table 1). The Slug expression level only showed a significant decrease in the IRS scoring system, and Twist showed no statistically significant difference between the placebo and CXB groups.

**Table 1:** Expression level of upstream EMT signalling in the tumour xenograft mice model by IHC combined semiquantitative scoring systems.

Maker	Placebo							CXB								
	66T	44T	54T	57T	69T	65T	50T	49T	61T	55T	48T	71T	58T	37T	p value^†^	
**Membrane EGFR**																
Intensity	2+	−	+	−	2+	+	2+	−	−	+	−	−	−	+		
Percentage	85	0	80	0	40	10	70	0	0	10	0	0	0	30		
Allred Score	7	0	6	0	6	3	7	0	0	3	0	0	0	4	0.0322	
IRS Score	8	0	3	0	4	1	6	0	0	1	0	0	0	2		0.0326
**Nuclear FAK**																
Intensity	+	+	+	−	+	+	+	−	−	−	+	−	+	+		
Percentage	80	60	60	0	90	60	50	0	0	0	30	0	50	30		
Allred Score	6	5	5	0	6	5	5	0	0	0	4	0	5	4	0.0113	
IRS Score	4	3	3	0	4	3	2	0	0	0	2	0	2	2		0.0100
**Nuclear Slug**																
Intensity	2+	+	+	−	+	−	+	−	−	−	−	−	−	2+		
Percentage	90	20	70	0	50	0	40	0	0	0	0	0	0	70		
Allred Score	7	4	6	0	5	0	5	0	0	0	0	0	0	7	0.0514	
IRS Score	8	2	3	0	2	0	2	0	0	0	0	0	0	6		0.0439
**Nuclear Twist**																
Intensity	+	−	−	−	−	+	+	−	−	−	−	−	+	−		
Percentage	70	0	0	0	0	80	80	0	0	0	0	0	30	0		
Allred Score	6	0	0	0	0	6	6	0	0	0	0	0	4	0	0.0986	
IRS Score	3	0	0	0	0	3	3	0	0	0	0	0	2	0		0.0986
**Nuclear ZEB1**																
Intensity	+	+	−	−	−	+	+	−	−	−	−	−	+	+		
Percentage	60	10	0	0	0	60	60	0	0	0	0	0	30	30		
Allred Score	5	3	0	0	0	5	5	0	0	0	0	0	4	4	0.0497	
IRS Score	3	2	0	0	0	3	3	0	0	0	0	0	2	2		0.0422

^†^The p value was calculated by the Wilcoxon signed-rank test.

### CXB use in decreasing the risk of oral cancer occurrence

The characteristics of subjects with and without CXB prescription on early prevention of oral cancer risk during the 13-year follow-up are demonstrated in Table 2 and Supplementary Table 1. The general risk factors of age and gender were significantly, independently associated with oral cancer occurrence (p < 0.0001). In Group 1 for oral cancer, the Cox proportional hazard regression analysis revealed subjects with CXB medication had an approximately 49% reduced risk of developing oral cancer [hazard ratio (HR) = 0.51, 95% CI, 0.42–0.62] compared with those who never used CXB. CXB use had a significant dose-response in the early prevention of oral cancer occurrence (*p* for linear trend < 0.0001), especially in subjects who had a daily dose of 100/200 mg (HR = 0.55, 95% CI, 0.37–0.81) or 200 mg (HR = 0.28, 95% CI, 0.20–0.40) of CXB. In the time-dependent effect analysis, subjects with more than 5 years of CXB medication had a significant reduction in oral cancer risk in Group 1 (HR = 0.33, 95% CI, 0.22–0.48) compared to those without CXB treatment. The increased period of CXB use also showed a significant time-dependent trend (*p* for linear trend <0.0001). Additionally, in oral cancer, including malignant neoplasm of oropharynx, hypopharynx and pharynx (Group 2), there was a 39% reduced risk in subjects with CXB use (HR = 0.61, 95% CI, 0.52–0.72). Only subjects with 200 mg of daily CXB had significant prevention of oral cancer (HR = 0.37, 95% CI, 0.28–0.48), and subjects with more than 5 years of CXB treatment had a preventive effect (HR = 0.29, 95% CI, 0.21–0.42), which was also the case for those in Group 1.

**Table 2:** CXB use associated with early prevention of oral cancer (Group 1) occurrence in a retrospective cohort study.

Characteristic	Without CXB use	With CXB use	p value
	n (%)	n (%)	
Total subjects	554965	49209	
Gender			
Male	275117 (49.57)	18067 (36.71)	<0.0001
Female	279848 (50.43)	31142 (63.29)	
Age (years)			
18–39	356630 (64.26)	5864 (11.92)	<0.0001
40–59	163516 (29.46)	22661 (46.05)	
≥60	34819 (6.27)	20684 (42.03)	
Oral cancer			
No	553256 (99.69)	49094 (99.77)	0.0004
Yes	1709 (0.31)	115 (0.23)	
ICD-9-CM			
140	139 (8.13)	7 (6.09)	
141	493 (28.85)	40 (34.78)	
143	107 (6.26)	5 (4.35)	
144	43 (2.52)	1 (0.87)	
145	927 (54.24)	62 (53.91)	
Preventive effect based on prescription record of CXB use^†^	1.00	0.51 (0.42–0.62)^*^	
Dose-dependent effect			
Dose (100 mg daily)	1.00	1.01 (0.77–1.34)	
Dose (100/200 mg daily)	1.00	0.55 (0.37–0.81)^*^	
Dose (200 mg daily)	1.00	0.28 (0.20–0.40)^*^	
		P_trend_ < 0.0001	
Time-dependent effect^‡^			
1–3 years	1.00	0.84 (0.66–1.07)	
3–5 years	1.00	0.65 (0.38–1.13)	
≥5 years	1.00	0.33 (0.22–0.48)^*^	
		P_trend_ < 0.0001	

^†^Hazard ratio was examined using a Cox proportional hazard model with adjustment for the age and gender in a cohort study followed up from 1997 to 2010.

^‡^Follow-up years after the first CXB use.

^*^p < 0.01.

## Discussion

The specific aim of this study is to evaluate the chemopreventive effect of CXB repositioning use against cancer occurrence and OSCC progression. An increased COX-2 level was observed in well to moderately differentiated tumours and in metastases^23^. Whether CXB use both decreases OSCC cell proliferation and inhibits cancer cell motility and EMT programs through COX-2 inhibition is worthy of attention. Based on the findings in this study, we suggest that clinical CXB use has a potential benefit for preventive and therapeutic implementation in subjects with a high risk of developing OSCC and in patients at an early stage who lack nodal metastases.

In preclinical models, significant inhibition of HNSCC tumour growth by COX-2 blockade using CXB has previously been reported^24^; also, we had *in vitro* findings detected by PCNA, Ki-67 and p-ERK1/2 in OSCC cells. The possible molecular mechanism may involve activation of the Ras/ERK1/2 mitogen-activated protein kinase pathway to elicit cell cycle arrest by inducing the cyclin-dependent kinase inhibitors, p21(WAF) and p27(kip1), to inhibit PCNA and Ki-67 in cancer cell proliferation^24^. We also found that an overexpression hallmark of many carcinomas, FOXM1, was significantly inhibited by CXB in OSCC cells. FOXM1 is a transcription factor driving the FOXO-dependent genes in regulation of cancer cell cycle progression and proliferation as well as in promotion of angiogenesis, EMT signalling, and cancer cell migration and invasion^25^. In addition, our murine xenograft model showed a 65% reduction in OSCC tumour growth, and the pathohistological status of xenograft tumours tended to be well-differentiated after 5-week treatments of CXB (100 mg/Kg/day) by gelatine-based oral gavage feeding.

The EMT programs involve several signalling pathways. Here, we showed several biomarkers for EMT progression in OSCC cells were reversible by CXB treatment, such as the inhibition of important nuclear transcriptional factors (Slug, Snail, Twist and ZEB1) and the subsequent initiation of cadherin switch (E-cadherin and N-cadherin) and cytoplasmic downstream signalling^15, 26^. The E-Cadherin/β-catenin complex is well-known to maintain cell-cell junctions of epithelial integrity. The decreased expression level of membranous β-catenin was found at the invasive front of well- and moderate-differentiated basal cell carcinoma of the head and neck, indicating invasive cellular behaviour^27^. A possible molecular mechanism was explored wherein the cytoplasmic tail of E-cadherin was liberated by cytosolic β-catenin fractions following loss of E-cadherin, leading to translocation of free β-catenin into the nucleus, where it can bind to Tcf/Lef transcriptional factors and activate the transcription of c-Myc, cyclin D1 and matrix metalloproteinase for EMT programs and invasion^28^. Recently, it was reported that the mRNA expression levels of CDH-1 (E-cadherin) and EMT transcriptional factors (Snail, Twist and ZEB2) were significantly induced and suppressed in the OSCC cell lines (HSC-2 and HSC-4), respectively, after 50 μM of CXB treatment for 12 h^29^. Moreover, it showed the lowering CDH-1 mRNA expression may increase risk in affecting lymph node metastasis. Our findings in HSC-3/HSC-3-RFP cell models showed that 100 μM of CXB treatment can block several EMT-related markers and raise E-cadherin at the protein expression level. Based on these findings, the CXB treatment may have potential effects in the early prevention of OSCC cancer metastases.

Regarding the overexpression level of COX-2 in most HNSCC tumour tissues, PGE_2_ was shown to induce “inside-out” integrin signalling through fibronectin (FN) ligand, α_5_β_1_ integrin and FAK activation toward tumour progression of EMT, migration and invasion^30^, while “outside-in” integrin signalling through FN, α_5_β_1_ integrin and FAK phosphorylation can induce COX-2 overexpression and PGE_2_ synthesis toward anti-apoptosis and tumour proliferation^31^. Endogenous FN can be produced by squamous epithelial cells obtained from upper aerodigestive tract cancers and then secreted out as exogenous FN^32^. Furthermore, it was reported that soluble FN containing an alternatively spliced extra domain A (EDA-FN) that was mostly derived from tumours or transformed cells can promote OSCC cell migration during cancer progression compared with an alternatively spliced extra domain B (EDB-FN) that was secreted from normal human fibroblasts^33, 34^. The endogenous level of FN in both HSC-3 and HSC-3-RFP cells was decreased after CXB treatment; however, we did not further confirm the FN isoform in this study. The integrin α_v_β_3_ hetero-dimers conferred metastatic progression and angiogenesis, and the molecular mechanisms were involved in the Ras/FAK-Raf-ERK signalling pathways through different activation of basic fibroblast growth factor (bFGF)/α_v_β_3_^35^. The increased FAK mRNA level and dysregulation of integrin-FAK signalling in HNSCC leads to cancer cell migration and invasion^36^; recently, it was shown that overexpression of nuclear FAK in SCC can promote tumour evasion by regulating inflammatory chemokine transcription and inducing an immune-suppressive microenvironment^37^. Our *in vivo* model showed that CXB treatment significantly inhibited both overexpression of nuclear Slug and ZEB1 and nuclear FAK in the xenograft OSCC tumours. The findings indicated that CXB treatment may decrease OSCC tumour tolerance and metastasis through inhibiting upstream signalling of the EMT.

Cancer cell motility and invasion are prerequisites for tumour metastasis, which necessitates rearrangement of the F-actin cytoskeleton. The Arp2/3 complex was reported to be associated with integrin adhesions^38^, and it was proposed that the Arp2/3 complex is recruited into focal adhesions by FAK^39^. In addition, the Arp2/3 complex was activated by a nucleation-promoting factor, cortactin, at the cell surface for regulating the polymerization of actin during cell motility^40^. Approximately 30% of HNSCC patients had cortactin overexpression, and this increased binding and activation of Arp2/3 complex triggered cancer cell motility and a more invasive phenotype^41^. Recently, the functional role of the Arp2/3 complex is proposed as a mechanism for controlling tumour cell motility, and it is suggested that inhibition of Arp2/3 activity may also provide new preventive therapies for invasive and metastatic cancers^42^. In addition, a variety of tumour-secreted factors can stimulate tumour cell mobility, such as autocrine motility factor (AMF), which is one of the important tumour motility-stimulating glycoproteins secreted by malignant tumour cells that binds to autocrine motility factor receptor (AMFR) gp78 to alter motility and invasion^43^. Overexpressed AMF and AMFR in OSCC cells were reported to increase the invasiveness and metastatic potentials^44^. A possible mechanism showed that AMF-induced motility involved the loss of E-cadherin through the upregulation of Snail protein^45^.

The EGFR-mediated signalling pathways are well-characterized in cell cycle progression, proliferation, invasion, and metastasis. Highly expressed mRNA of EGFR was detected in more than 90% of histologically normal mucosa and cancerous parts excised from HNSCC patients compared with control samples from non-cancer patients^46^. An elevated expression level of EGFR resulted in poor overall and relapse-free survivals in HNSCC patients^47^. This implies that the marginal normal mucosa in HNSCC patients still has a high risk of developing recurrent malignancy after standard surgery because of the high expression level of EGFR. Moreover, the evidence suggests that cross-talk among the signalling cascades of COX-2-derived PGE_2_, loss of E-cadherin and EGFR activation enhances the cancer cell proliferation, EMT program and metastasis^48–50^. Although target drug therapy using an anti-EGFR monoclonal antibody, cetuximab, has been approved in the clinical treatments for patients with locally or regionally advanced HNSCC^51, 52^ and platinum-refractory, recurrent or metastatic HNSCC^52^, the burden of medical costs for many patients is considered. In this study, we showed that CXB treatment significantly decreased the EGFR expression in OSCC using *in vitro* and *in vivo* models. Here, we suggest that combination therapy with a low-cost routine medication, CXB, after surgery may provide an alternative option for relapse-free OSCC patients in preventing cancer recurrence and progression.

In our *in vitro* findings, CXB caused different alterations of the expression level of some detected biomarkers between original HSC-3 and established HSC-3-RFP cell lines. Masood *et al*. reported that the establishment of US-HN3-GFP-G2 stable cell lines (generation 2) showed significantly higher gene expression levels of several biomarkers when compared with US-HN3-GFP-G1 stable cell lines (generation 1) and original USC-HN3 cell lines^53^. However, the inconsistent findings between the original and established inflorescent stable cell lines remain to be completely elucidated. In an *in vivo* study, the orthotopic xenograft model is the precise method for investigating the mechanism of regional or distant metastasis in head and neck cancer compared with a subcutaneous ectopic model; however, it can be more technically challenging to establish this model because of the higher mortality and lower success rate of xenotransplantation in mice^54^. Moreover, an orthotopic model of oral cancer in the dorsal tongue xenograft in nude mice resulted in dysphagia and weight loss^55^. The symptoms may affect the observation of the major side effect of CXB in this study. To date, a few studies have been successfully performed using an orthotopic model for oral cancer, and several experimental skills need to be adjusted and established in this model. Although the subcutaneous ectopic models cannot mimic metastasis from human oral cancer, the absorption and distribution of CXB through oral gavage in the murine body and effect in decreasing upstream EMT signalling has reference value in oral cancer prevention. In the population-based study, the strengths of the study design were described below. First, the selection and recall biases were reduced by employing a cohort design. Subsequently, the rate of loss to follow-up was low due to its compulsive characteristics, high coverage of the entire population and management by the government. However, the limitations include that some information is not available in the surveillance database, which may influence the evaluation and interpretation of our findings, including the use of substances such as alcohol, betel quids and cigarettes as well as the actual CXB use after prescription.

**Figure 4:**
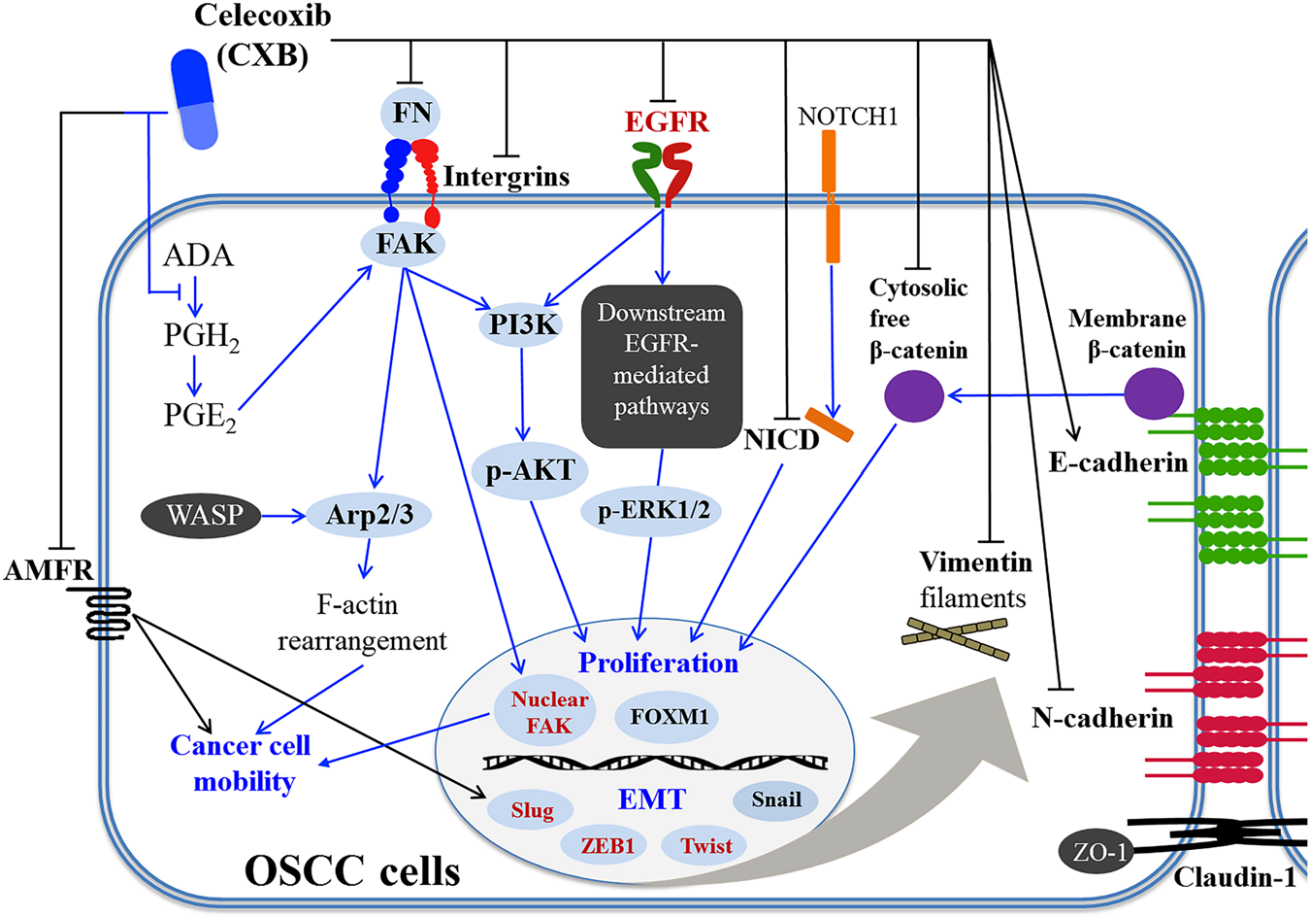
The possible molecular mechanism of CXB in the chemoprevention of OSCC cancer progression. The protein levels of selected biomarkers in bold were investigated in the cell models, and the four biomarkers in red were further analysed in a mouse xenograft model.

In summary, the present study provides valuable information about CXB use in inhibiting OSCC EMT programs and cancer cell mobility by *in vitro* and *in vivo* models (Fig. 4). In addition, based on an epidemiological evaluation from a population-based study, we speculate that CBX use has a potential effect in the early prevention of OSCC occurrence in a dose- and time-dependent manner. Currently, we have an ongoing intervention study (phase II trial) to investigate the effect of CXB medication on recurrence prevention and improved prognosis in relapse-free OSCC patients (ClinicalTrials.gov Identifier: NCT02739204). The therapeutic and preventive strategies of ready-to-use CXB in OSCC patients will be thoroughly evaluated as a new indication based on the evidence in this preclinical study and further clinical trials.

## Methods

### Cell culture and CXB

SCC-9 (CRL-1629), CAL 27 (CRL-2095, ATCC, USA), HSC-3 (JCRB0623), SAS (JCRB0260), Ca9-22 (JCRB0625, JCRB Cell Bank, Japan) and 2 primarily cultured OSCC cells (BQO and ABC) were used in the study. BQO and ABC cells were established from a 55-year-old male patient with gingival squamous cell carcinoma (stage IVa, T_2_N_2_M_0_) and a 40-year-old female patient with tongue squamous cell carcinoma (stage IVa, T_2_N_0_M_x_), respectively. The informed consent was obtained from OSCC patients. All methods were performed in accordance with relevant guidelines and regulations, and all experimental protocols were approved by the Institutional Review Board Committee, Changhua Christian Hospital, Taiwan (IRB No. 090513). All OSCC cells were cultured in DMEM/F-12 medium supplemented with 10% foetal bovine serum (FBS), 40 ng/mL hydrocortisone, 0.1 mM non-essential amino acids solution, and a mixture of 100 U/ml penicillin and 0.1 mg/mL streptomycin (PS) (Gibco, Thermo Fisher Scientific). The human papillomavirus (HPV) infection status was determined using a hc2 High-Risk HPV DNA Test (Qiagen). The value of the RLU/Cut-off less than 1.0 indicated that cells had no infection with 13 high-risk HPVs (types 16, 18, 31, 33, 35, 39, 45, 51, 52, 56, 58, 59 and 68). CXB (Pfizer) was purchased from a local pharmacy. The powder of CXB was dissolved and diluted in dimethyl sulphoxide (DMSO; Hybri-Max, Sigma-Aldrich) at appropriate concentrations for cell models. CXB, mixed with a 10% sterilized gelatine solution (Merck), was used for murine gavage feeding.

### Cell viability

OSCC cells were seeded into 96-well plates and incubated overnight, which was followed by treatments with a serial concentration of CXB (0, 25, 50, 100 and 200 µM) for 12, 24, and 48 hours. The final concentration of DMSO was 0.1% in all treatments. Cell viability (%) was examined using an *in vitro* toxicology assay kit (MTT based, Sigma-Aldrich).

### Proliferation assay

Total protein lysates from OSCC cells were harvested after 24 h of CXB treatment (0, 25, 50 and 100 µM) using a RIPA lysis buffer (Cell Signaling Technology). Briefly, the samples were examined using anti-PCNA (GeneTex) normalized by β-actin (Millipore) in an immunoblotting analysis. In an immunocytochemistry and immunofluorescence (ICC-IF) assay, OSCC cells were seeded on glass slides coated with poly-L-lysine (Sigma-Aldrich) prior to 24 h treatments (0 and 100 µM). Cells were then fixed with 4% paraformaldehyde (Merk) and blocked with 10% normal goat serum containing 0.3% of Triton X-100, followed hybridization of anti-Ki-67 (Cell Signaling Technology) and a secondary fluorescent antibody conjugated with Alexa Fluor®488 (Thermo Fisher Scientific). The ProLong Gold Antifade Mountant with DAPI nuclear stain (Thermo Fisher Scientific) was applied to the slides prior to visualization under a fluorescence microscope (DM4000 M, Leica Microsystems).

### Scratch migration assay

The scratch-wound assay was performed to test the inhibitory effect of CXB in OSCC cell migration. OSCC cells were seeded into 12-well plates and cultured to 90% confluence. A cell monolayer was scratched with a 1-mL micropipette tip in each well, and treated with CXB (0, 25, 50 and 100 μM) for 0, 12 and 24 hours. The migration ability was captured under a microscope (DM IL, Leica Microsystems), and the relative migration rate was quantified with Image J software (http://imagej.nih.gov/ij/).

### Transwell migration and invasion assay

In the migration assay, OSCC cells were mixed with cultured medium containing 0.5% FBS and seeded into the upper chambers of the inserts with an 8-μm pore size of polyethylene terephthalate membrane (Millipore). The inserts were placed in 24-well plates containing complete medium with a series concentration of CXB (0, 25, 50 and 100 μM) in lower wells, respectively. The cell number ranged from 1 × 10^5^ to 2 × 10^6^ cells to evaluate the diversity characteristics of the migration or invasion activity among OSCC cells. After 24 h of treatment, the inserts were fixed in 4% paraformaldehyde. The top-chamber cells were removed by swabbing and migrated cells were stained with 0.1% crystal violet (w/v) in 20% methanol (v/v). The migration ability was observed and captured under a microscope (DM IL, Leica Microsystems). The invasiveness of treated OSCC cells (0, 25, 50 and 100 μM) was determined using a BioCoat Matrigel Invasion Chamber (Corning) set according to the Cell Invasion Assay protocol (http://csmedia2.corning.com/LifeSciences/media/pdf/protocol_DL_031_Cell_Invasion_Assay.pdf).

### Immunoblotting

Western blot analysis was performed using an Epithelial-Mesenchymal Transition Antibody Sampler kit (N-cadherin, E-cadherin, Vimentin, Claudin-1, β-catenin, Snail, Slug and TCF8/ZEB1 antibodies), Integrin Antibody Sampler Kit (Integrin α4, α5, αV, β1, β3, β4 and β5 antibodies), anti-FAK, anti-PI3K, anti-AKT, anti-p-AKT (Ser473), anti-ERK1/2, anti-p-ERK1/2, anti-Notch1, anti-EGFR (Cell Signaling Technology), anti-Twist, anti-fibronectin (FN) (Abcam), anti-FOXM1, anti-Arp2, anti-Arp3, anti-AMFR (GeneTex), and anti-GAPDH antibodies (Millipore). The chemiluminescent signals were visualized and quantified using ImageQuant LAS 4000 with ImageQuant LAS 4000 Biomolecular Imager software (GE Healthcare). The difference in the protein expression level between treated and untreated cells was analysed in at least 3 independent experiments.

### Murine xenograft tumour model

The OSCC cell line that we selected in this model was HSC-3, which showed 30% metastatic potential to lymph nodes in 3 weeks after injection into the tongue in a murine model^32^. The lentiviral system with a stably expressed red fluorescent protein (RFP) in HSC-3 cells was established for the xenograft tumour model. HEK-293FT cells were transfected with lentivirus package plasmids (pCMV delta R8.91, pAS2.RFP and pMDG; RNAi Core Laboratory, Academia Sinica, Taiwan) using Lipofectamine 2000 reagent (Thermo Fisher Scientific) for an 18 h incubation. Culture medium using α-MEM with 10% FBS, 1% BSA, and 1% PS was replaced every 24 hours. The pseudo-typed lentiviral particles were collected in 3 days and filtered with a 0.45-μm membrane pore size (Millipore). A mixture of lentivirus supernatant and 10 μg/mL polybrene (Sigma Aldrich) was added to HSC-3 for 24 h of infection. HSC-3-RFP cells were selected using 2 μg/mL puromycin (Sigma-Aldrich) until the stable clone was completely established.

The dorsal subcutaneous skin of 16 5-week-old nude mice (BALB/c-nu/nu) was injected with 5 × 10^6^ of HSC-3-RPF cells mixed with 100 µL of Matrigel (Corning). The signal intensity of tumour growth was monitored every week using an IVIS Imaging System (PerkinElmer) before grouping. The quantification of the tumour fluorescence signal based on total radiant efficiency per second with appropriate background subtraction was determined at the endpoint using a Living Image *In Vivo* Imaging Software (PerkinElmer). Mice were randomly assigned to the treated (n = 8) and control (n = 8) groups based on the xenograft tumour fluorescent signal. To reduce the residue of CXB in the syringe and gavage needle, we performed a gelatine-mixed method to implement the oral gavage feeding of water insoluble CXB. The treated group had an oral gavage feeding of 100 mg of CXB per Kg of body weight, while the control group received placebo (same volume of 10% of gelatine solution) for 5-week treatments (5 days a week). This was performed in accordance with the animal welfare guideline approved by China Medical University and Hospital (103-13-NH).

### Immunohistochemistry

Paraffin-embedded tissue sections (4 µm) of murine xenograft tumours were mounted on poly-L-lysine-coated glass slides. An immunohistochemistry-paraffin (IHC-P) staining protocol was implemented as described^32^, and primary antibodies of anti-Slug (1:50), anti-FAK (1:50), anti-EGFR (1:50) (Cell Signaling Technology), anti-Twist (1:200) and anti-ZEB1 (1:100) (Novus Biologicals) were used. IHC scoring was examined and discussed by two pathologists in the Department of Surgical Pathology, Changhua Christian Hospital, Taiwan (laboratory accreditation: College of American Pathologists, CAP# 7185748). Two widespread combinatory semiquantitative scoring systems for IHC evaluation were used, including the Allred-scoring system^56^ and immunoreactive scoring system (IRS)^33^.

### Nationwide population-based retrospective cohort study

The Taiwan National Health Insurance Research Database covers approximately 23 million people under the National Health Insurance Program (NHIP) in 1995. We obtained a cohort dataset of approximately 1 million people, who were followed up from 1997 to 2010, which was released from National Health Research Institutes (NHRI) in Taiwan. In our retrospective cohort design, subjects were classified into exposure and unexposed subjects according to the CXB prescription. Currently, there are inconsistent criteria for oral cancer in published papers due to the lack of consensus on the topography of the mouse region and the definition of oral cancer^57^. Therefore, at the endpoint of follow-up, we identified 1,824 subjects in Group 1 (ICD-9 codes: 140, 141, 143, 144 and 145) and 2,334 patients in Group 2 (ICD-9 codes: 140, 141, 143, 144, 145, 146, 148 and 149) who were newly diagnosed oral cancer according to the International Classification of Diseases, Ninth Revision, Clinical Modification (ICD-9-CM). The flow chart of this study design is summarized in Supplementary Fig. 4. The database provided de-coding secondary information. The data were analysed anonymously, and the requirement for informed consent was waived by ethical approval from the Institutional Review Board of Kaohsiung Medical University Hospital (KMUH-IRB-980174). All methods were performed and approved by KMUH and NHRI, Taiwan in accordance with relevant guidelines and regulations of NHIP.

### Statistical Analysis

The experimental data from *in vitro* and *in vivo* studies were analysed using the Student t-test, one-way ANOVA with Bonferroni correction, and simple regression model for trend test. The Wilcoxon signed-rank test was performed in the IHC analysis. A multivariate Cox proportional-hazards regression model was performed to evaluate the independent dose and time effect of CXB in prevention of oral cancer occurrence. All statistical analyses were conducted using SAS software (version 9.3, Cary, North Carolina, USA).

## Electronic supplementary material

Supplementary data
