# Supplementary material for: Preventive effect of celecoxib use against cancer progression and occurrence of oral squamous cell carcinoma
Source: Sci Rep. 2017 Jul 24;7:6235. doi: 10.1038/s41598-017-06673-3 (PMC5524966; doi:10.1038/s41598-017-06673-3)
Supplement: Supplementary file 1 — Supplementary data [file 41598_2017_6673_MOESM1_ESM.pdf]

Preventive effect of celecoxib use against cancer progression and occurrence of oral squamous cell carcinoma

Shang-Lun Chiang, Bharath Kumar Velmurugan, Chia-Min Chung, Shu-Hui Lin, Zhi-Hong Wang, Chun-Hung Hua, Ming-Hsui Tsai, Tzer-Min Kuo, Kun-Tu Yeh, Pei-Ying Chang, Yi-Hsin Yang, Ying-Chin Ko

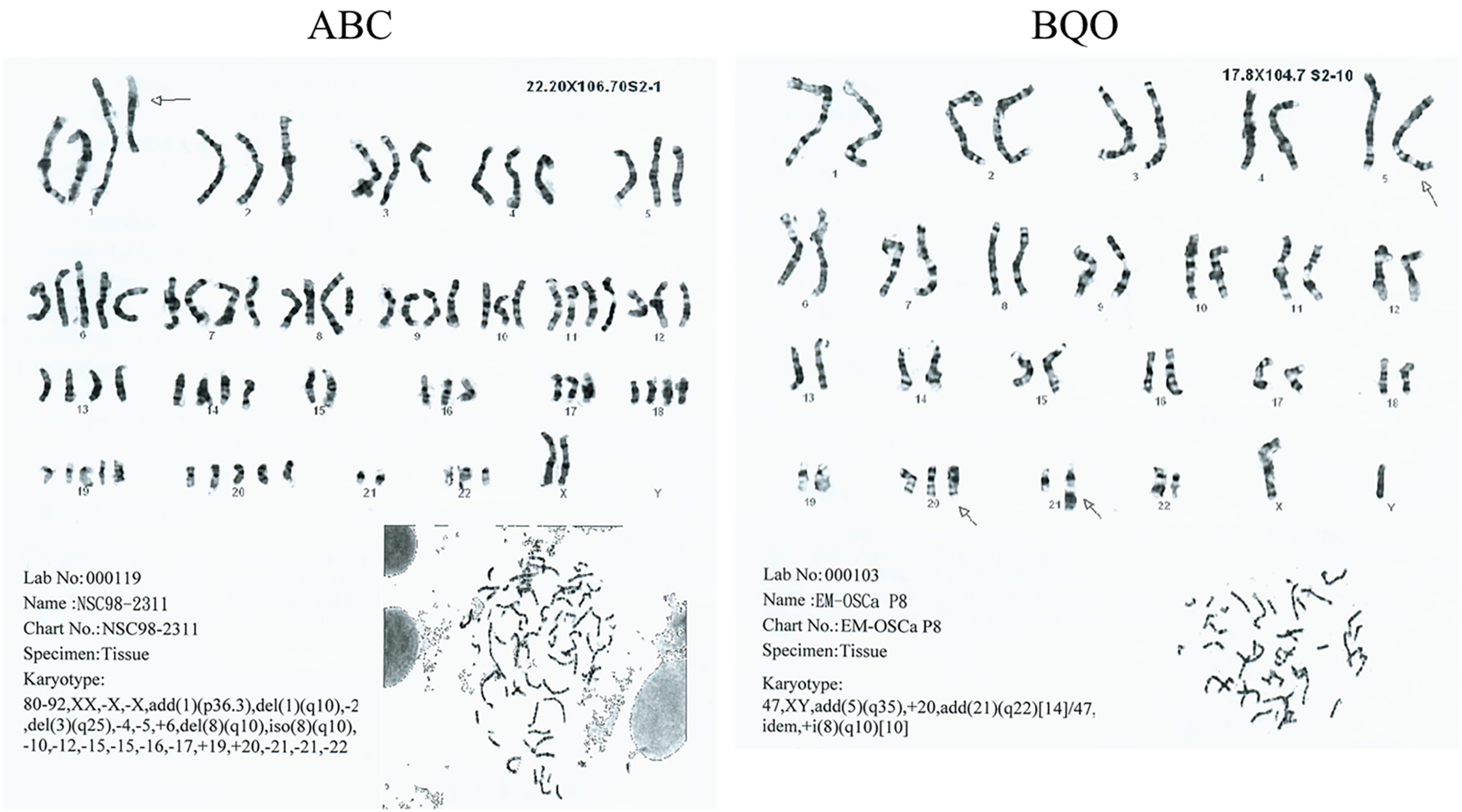

Supplementary Fig. 1. Cytogenetic analysis of two primarily cultured OSCC cells, ABC and BQO.

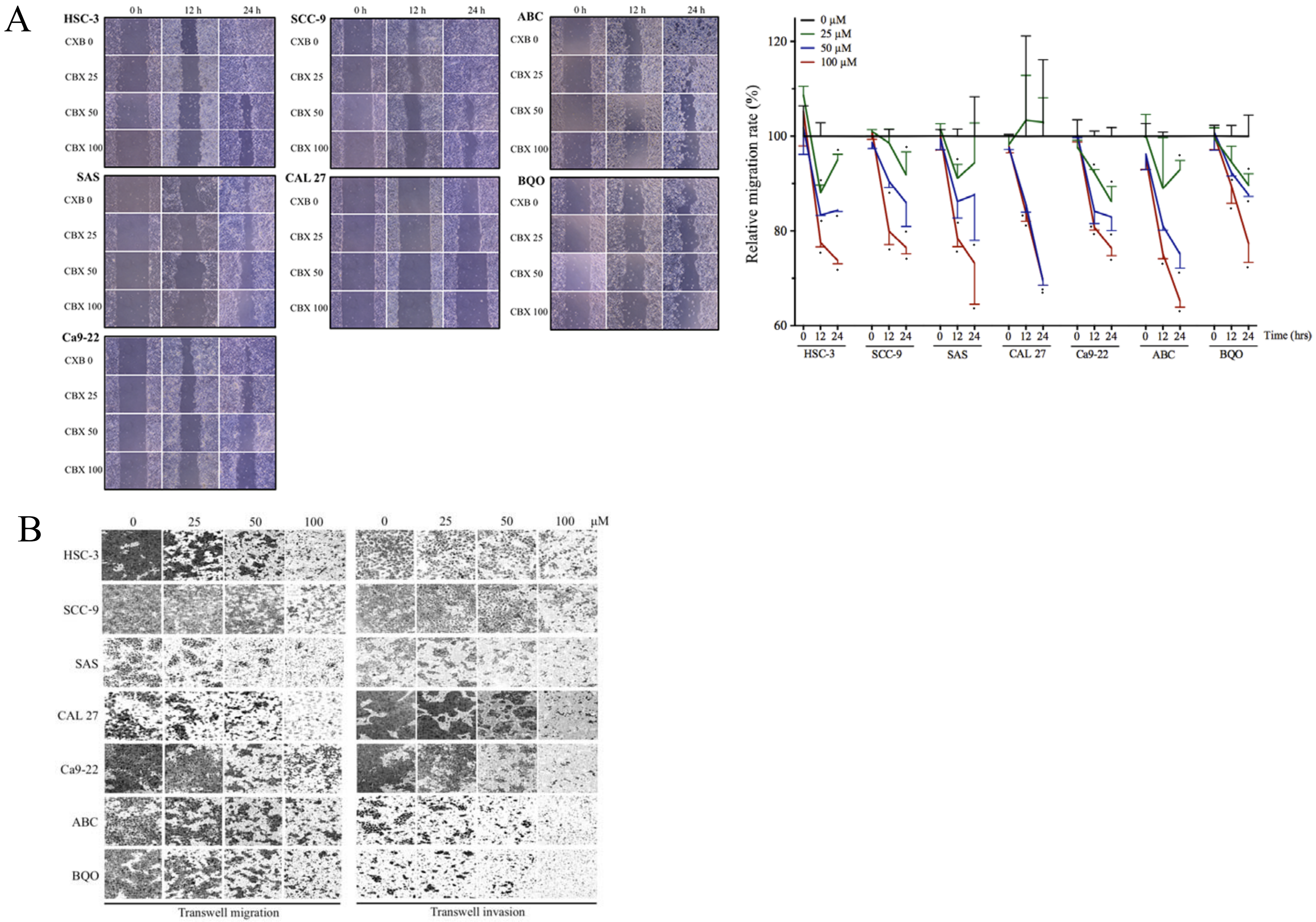

Supplementary Fig. 2. Determination of CXB effect on OSCC cell migration by scratch-wound and transwell assays and cell invasion by a matrigel-transwell assay.

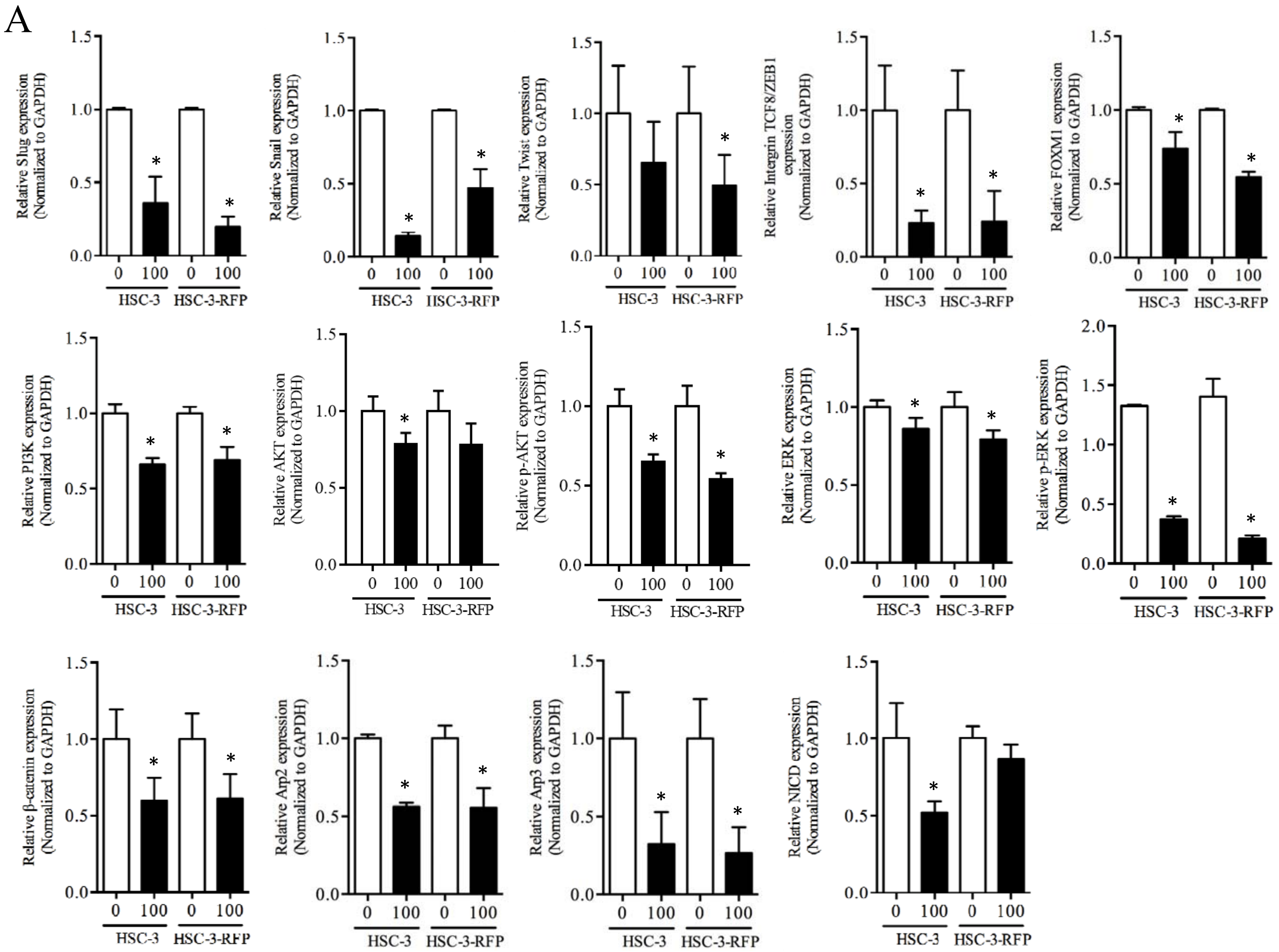

Supplementary Fig. 3. The CXB effect on selected markers involving in the signalling pathways of OSCC cell proliferation, EMT programs and cancer cell mobility.

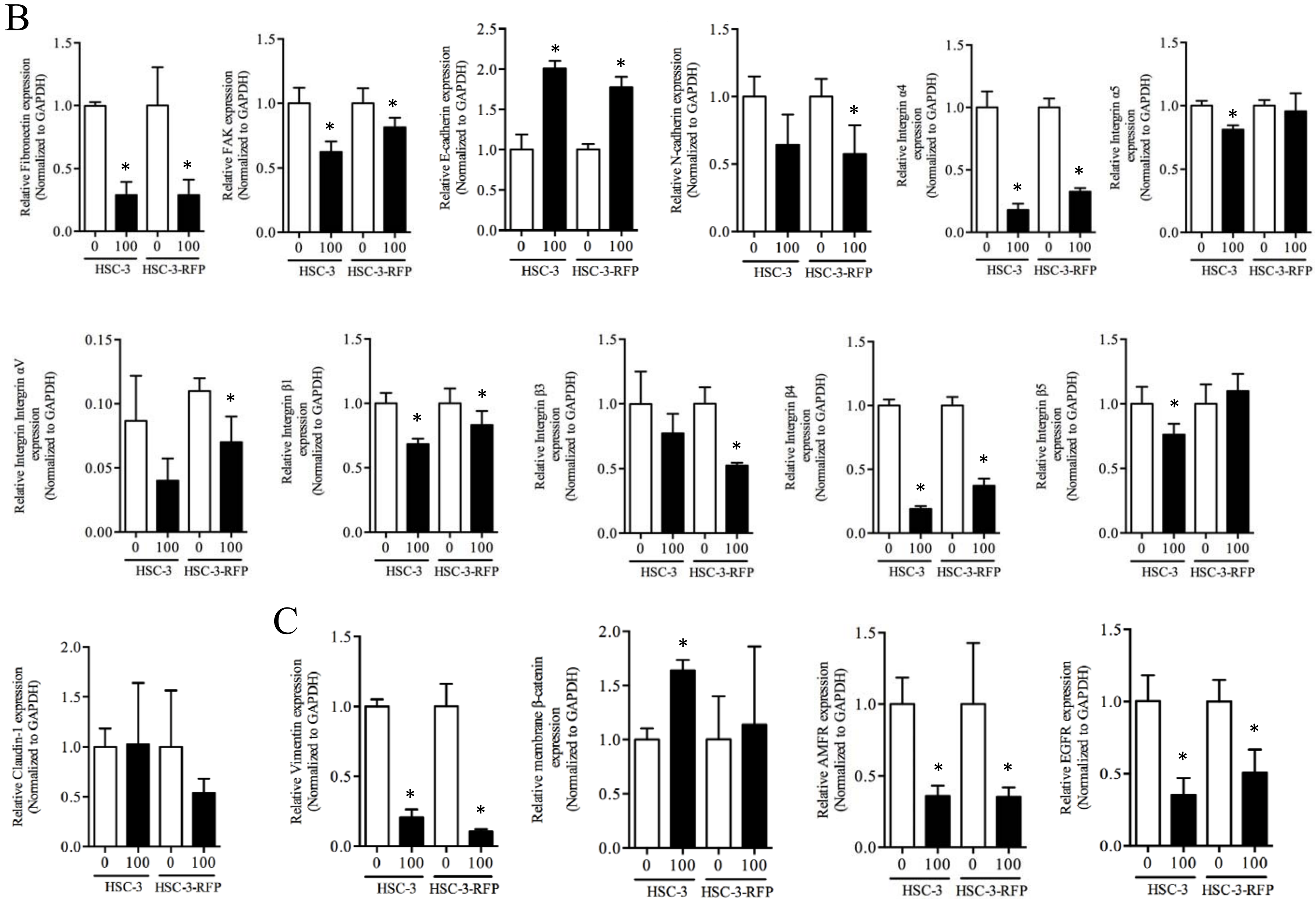

Supplementary Fig. 3. (continued)

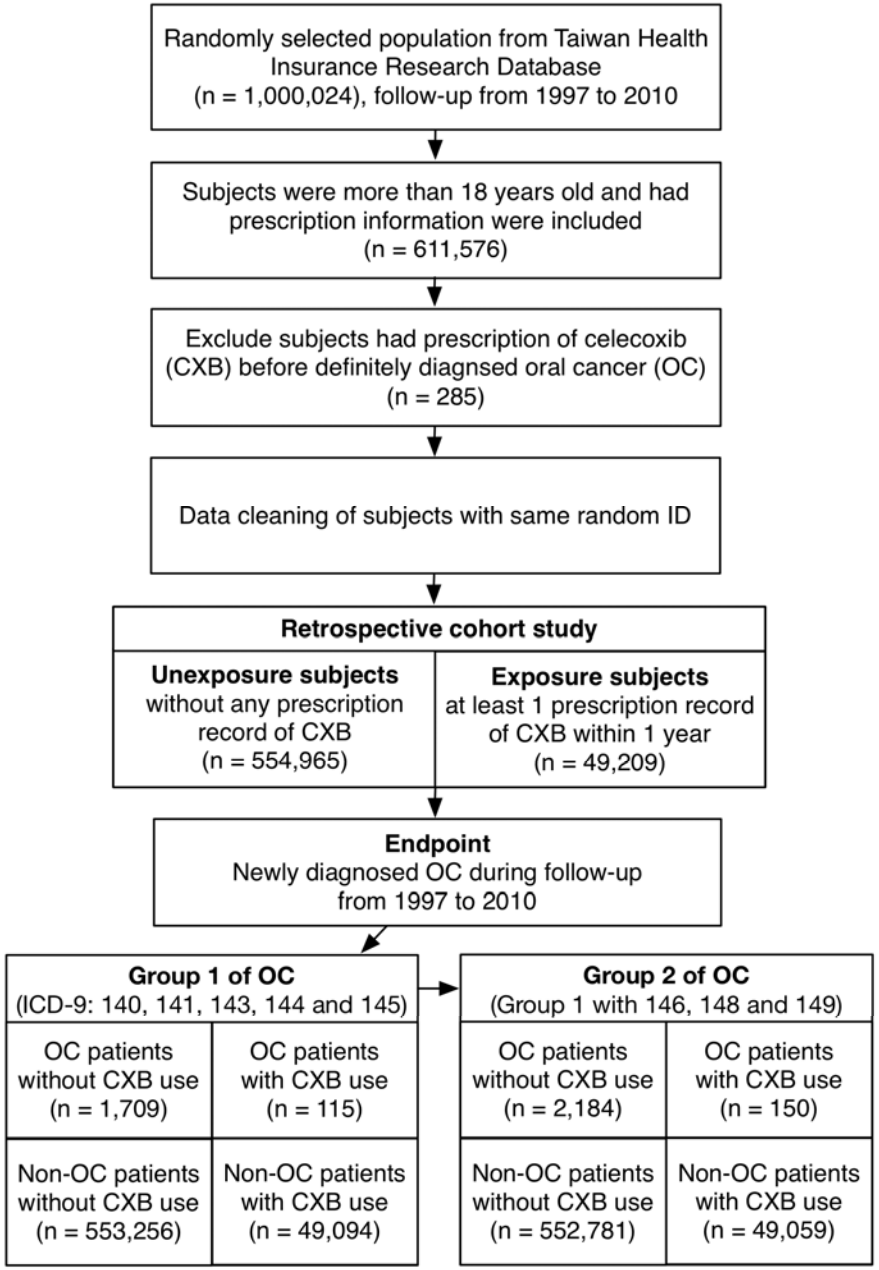

Supplementary Fig. 4. The flowchart describing the enrollment of subjects from National Health Insurance Research Database of Taiwan in this study.

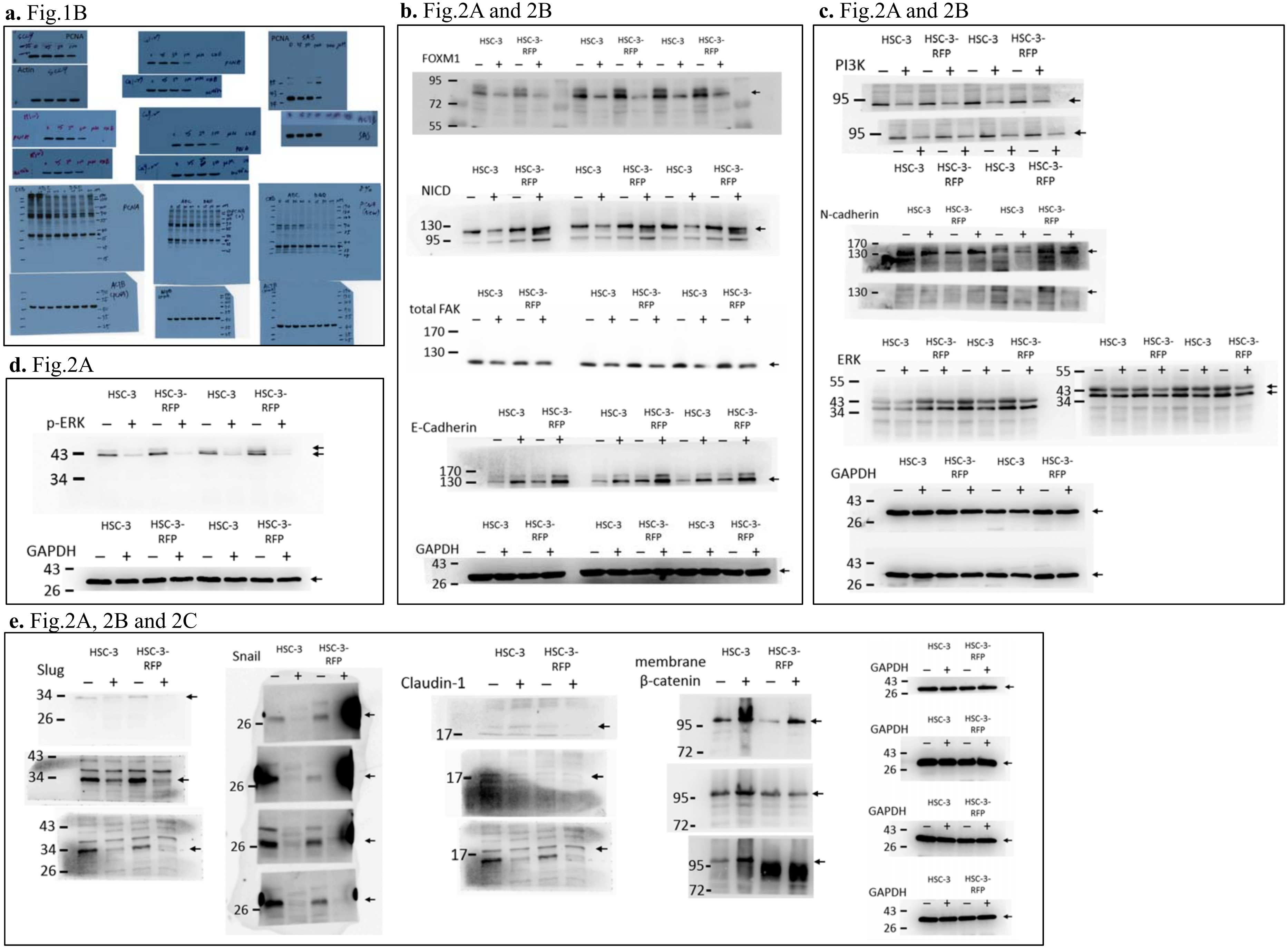

Supplementary Fig. 5. Original blots for all experiments. (a) Figure 1. (b)-(e) Figure 2.

f. Fig.2A, 2B and 2C

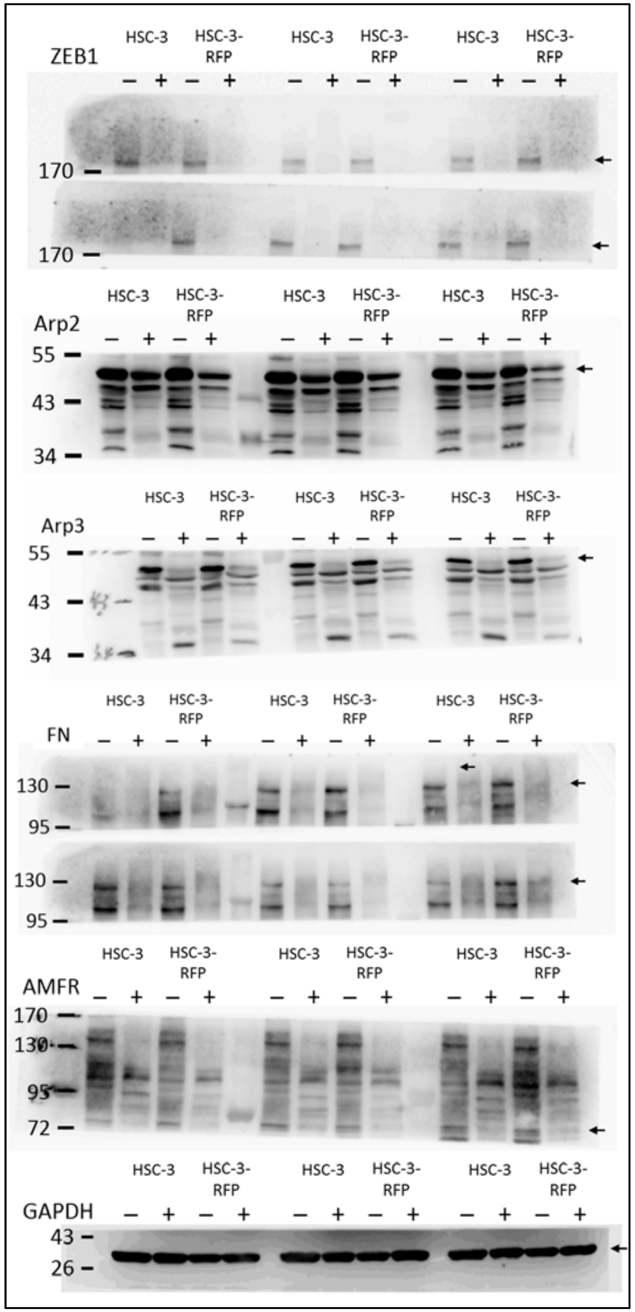

g. Fig.2B

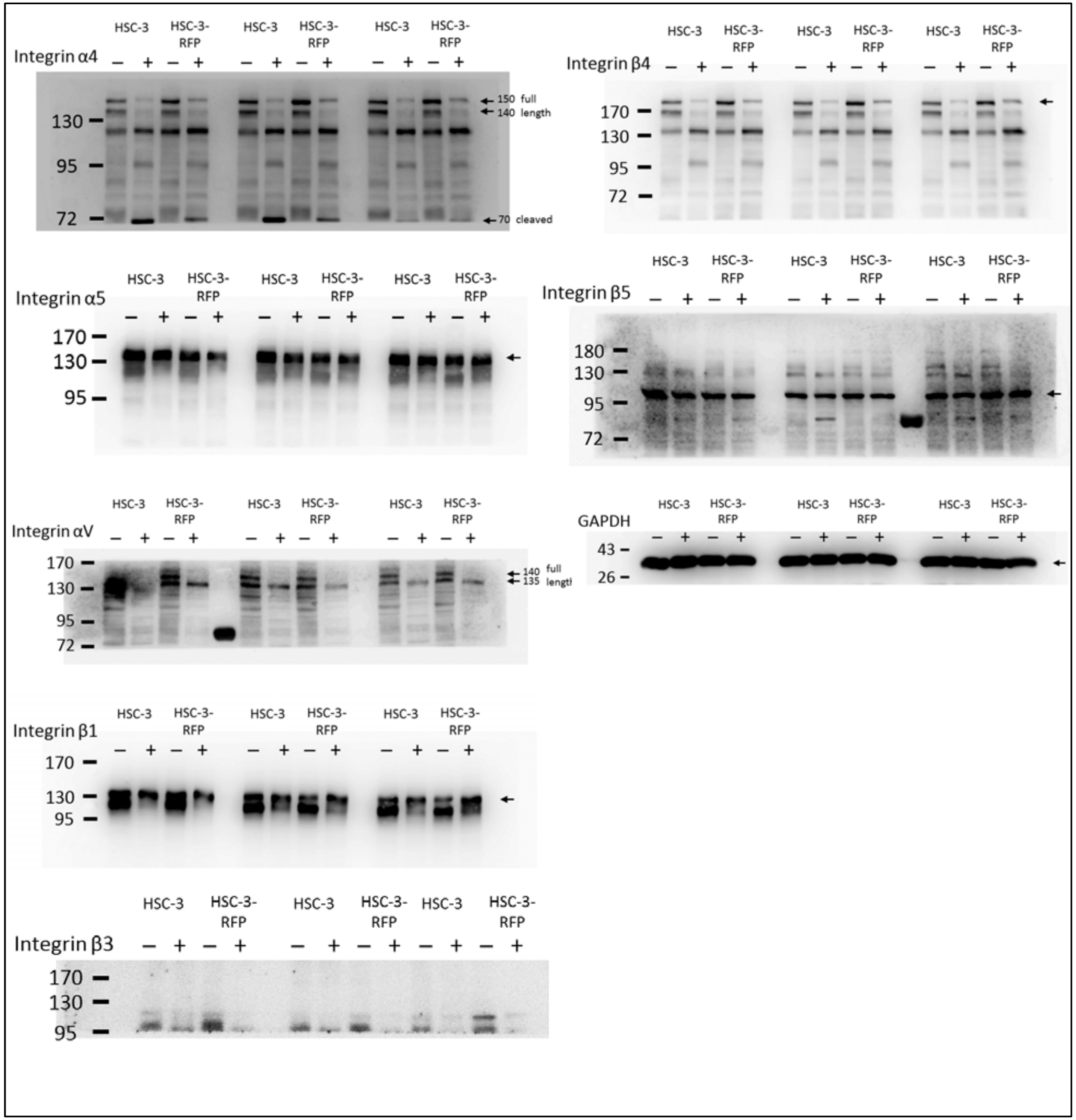

Supplementary Fig. 5. Original blots for all experiments (continued). (f) and (g) Figure 2.

h. Fig.2A and 2C

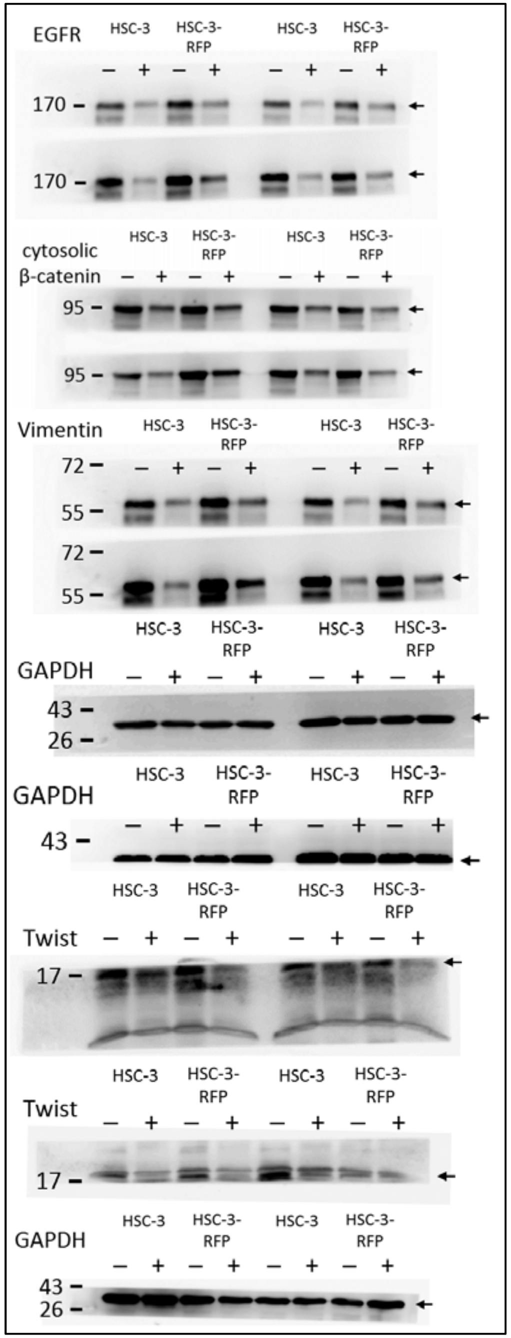

i. Fig.2A

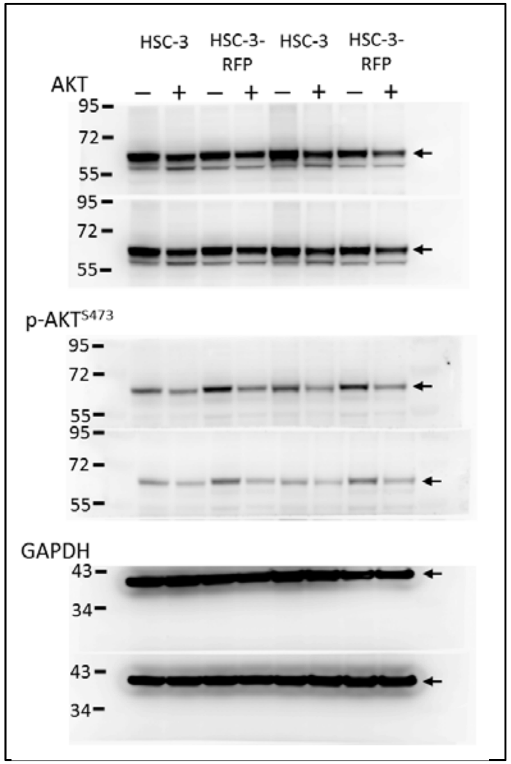

Supplementary Fig. 5. Original blots for all experiments (continued). (h) and (i) Figure 2.

Supplementary Table 1. CXB use associated with early prevention of oral cancer (Group 2) occurrence in a retrospective cohort study

Characteristic	Without CXB use n (%)	With CXB use n (%)	p value
Total subjects	554965	49209	
Gender			
Male	275117 (49.57)	18067 (36.71)	< 0.0001
Female	279848 (50.43)	31142 (63.29)	
Age (years)			
18 - 39	356630 (64.26)	5864 (11.92)	< 0.0001
40 - 59	163516 (29.46)	22661 (46.05)	
≥ 60	34819 (6.27)	20684 (42.03)	
Oral cancer			
No	552781 (99.61)	49059 (99.70)	< 0.0001
Yes	2184 (0.39)	150 (0.30)	
ICD-9-CM			
140	139 (6.36)	7 (4.67)	
141	493 (22.57)	40 (26.67)	
143	107 (4.90)	5 (3.33)	
144	43 (1.97)	1 (0.67)	
145	927 (42.45)	62 (41.33)	
146	204 (9.34)	13 (8.67)	
148	188 (8.61)	17 (11.33)	
149	83 (3.80)	5 (3.33)	
Preventive effect based on prescription record of CXB use†	1.00	0.61 (0.52-0.72)*	
Dose-dependent effect			
Dose (100 mg daily)	1.00	0.99 (0.77-1.26)	
Dose (100/200 mg daily)	1.00	0.79 (0.58-1.04)	
Dose (200 mg per daily)	1.00	0.37 (0.28-0.48)*	
		$P_{trend} < 0.0001$	
Time-dependent effect‡			
1 - 3 years	1.00	0.92 (0.76-1.12)	
3 - 5 years	1.00	0.86 (0.57-1.31)	
≥ 5 years	1.00	0.29 (0.21-0.42)*	
		$P_{trend} < 0.0001$	

†Hazard ratio was examined using a Cox proportional hazard model with adjustment for age and gender in a cohort study followed up from 1997 to 2010

‡Follow-up years after first CXB use

*, p < 0.01
